# Book Notices

**Published:** 1868-04

**Authors:** 


### Book Notices.

Treatise on Diseases of the Eye, Including the Anatomy of the Organ. By CARL STELLWAG VON CARION, M.D., Professor of Ophthalmology in the Imperial Royal University of Vienna. Translated from the Third German Edition, and edited by CHARLES E. HACKLEY, M.D., Surgeon to the New York Eye and Ear Infirmary, Physician to the New York Hospital, Fellow of the New York Academy of Medicine, etc., and D. B. ST. JOHN ROOSA, M.D., Clinical Professor of the Diseases of the Eye and Ear in the Medical Department of the University of the City of New York, etc. With an Appendix by the Editors. Illustrated by Ninety-Six Wood Engravings and Eighteen Chromo-Lithographs. New York: WM. WOOD & Co., 61 Walker Street. 1868.

This is a full-sized octavo volume of 774 pages, published in excellent style. There is not, within our knowledge, any one volume, accessible to the American student and practitioner, which affords so complete a view of the present state of knowledge in relation to the organs of vision and their diseases, medical and surgical, as the one before us. The editors and publishers have conferred a real favor on the profession. For sale by W. B. KEEN & Co., 148 Lake Street.

The Principles and Practice of Obstetrics. By GUNNING S. BEDFORD, A.M., M.D., Prof. of Obstetrics, the Diseases of

Women and Children, and Clinical Obstetrics in the University of New York; Author of Clinical Lectures on Diseases of Women and Children. Illustrated by Four Colored Lithograph Plates and Ninety-nine Wood Engravings. Fourth Edition, carefully revised throughout, and enlarged. New York: Wm. Wood & Co., 61 Walker Street. 1868.

The work on Obstetrics, by Prof. Bedford, is already so well known to the profession, that any comments in relation to its general merits are unnecessary. The present edition has been thoroughly revised, with the addition of some new matter. The publishers have done their part of the work well. We freely commend the work to the patronage of our readers, whether students or practitioners. For sale by medical booksellers generally.

---

A Practical Treatise on the Diseases of Women. By T. Gaillard Thomas, M.D., Prof. of Obstetrics and Diseases of Women and Children in the College of Physicians and Surgeons of New York; Physician to Bellevue Hospital; Consulting Physician to the State Women's Hospital, etc., etc. With Two Hundred and Nineteen Illustrations. Philadelphia: Henry C. Lea. 1868.

This is an octavo volume of 625 pages. Its typographical execution and binding are excellent; and we doubt not its contents are correspondingly interesting and valuable, though we have not yet commanded sufficient time to examine them in detail. The well-known ability and industry of the author will justify a full recommendation of the work. For sale by W. B. Keen & Co., 148 Lake Street.

---

Lectures on Orthopædic Surgery. Delivered at the Brooklyn Medical and Surgical Institute. By Louis Bauer, M.D., M.R.C.S.. Eng., Prof. of Anatomy and Clinical Surgery; Licentiate of the New York Medical Society; Member of the New York Pathological Society; Member of the American Medical Association; Corresponding Fellow of the London

Medical Society, etc., etc. Second Edition, revised and augmented, with Eighty-four Illustrations. New York: Wm. Wood & Co., 61 Walker Street. 1868.

This edition of Dr. Bauer's work embraces 336 octavo pages, being much larger than the first edition. The author is an earnest, thoughtful investigator and experienced practitioner; and our readers will find his work a valuable addition to their libraries. For sale by W. B. Keen & Co., 148 Lake Street.

